# In Situ Synthesis of Gold Nanoparticles on Wool Powder and Their Catalytic Application

**DOI:** 10.3390/ma10030295

**Published:** 2017-03-15

**Authors:** Bin Tang, Xu Zhou, Tian Zeng, Xia Lin, Ji Zhou, Yong Ye, Xungai Wang

**Affiliations:** 1 National Engineering Laboratory for Advanced Yarn and Fabric Formation and Clean Production, Wuhan Textile University, Wuhan 430073, China; shirleyliem@sina.com (X.L.); xungai.wang@deakin.edu.au (X.W.); 2 Hubei Collaborative Innovation Center for Advanced Organic Chemical Materials & Key Laboratory for the Synthesis and Application of Organic Functional Molecules, Ministry of Education & College of Chemistry & Chemical Engineering, Hubei University, Wuhan 430062, China; zhouxu@stu.huhu.edu.cn (X.Z.); czeng@jhlbiotech.com (T.Z.); yeyong@hubu.edu.cn (Y.Y.); 3 Institute for Frontier Materials, Deakin University, Geelong, Victoria 3216, Australia

**Keywords:** gold nanoparticles, wool powder, biomass complex, in situ synthesis, catalysis

## Abstract

Gold nanoparticles (AuNPs) were synthesized in situ on wool powder (WP) under heating conditions. Wool powder not only reduced Au ions to AuNPs, but also provided a support for as-synthesized AuNPs. WPs were treated under different concentrations of Au ions, and corresponding optical features and morphologies of the treated WPs were investigated by UV-VIS diffuse reflectance absorption spectroscopy and scanning electron microscopy (SEM). X-ray diffraction (XRD), X-ray photoelectron spectroscopy (XPS), and transmission electron microscope (TEM) were also employed to characterize the WP treated with AuNPs. The results demonstrate that AuNPs were produced in the presence of WP and distributed over the wool particles. The porous structure led to the synthesis of AuNPs in the internal parts of WP. Acid conditions and high temperature facilitated the synthesis of AuNPs by WP in aqueous solution. The reducibility of wool was improved after being converted to powder from fibers, due to exposure of more active groups. Moreover, the obtained AuNP-WP complexes showed significant catalytic activity to accelerate the reduction reaction of 4-nitrophenol (4-NP) by sodium borohydride (NaBH_4_).

## 1. Introduction

Wool powder (WP) is produced from fiber wastes in wool yarn and garment manufacture [1]. Different from bulk wool fibers, microscaled powders or particles show particular properties, such as porosity and absorptivity. Milling is one of effective methods to convert wool fibers to microstructural powders, which avoids long and costly chemical routes and use of harmful reagents [2]. Wool powders (WPs) from milling inherit the microstructures and component features of wool fiber. With regards to wool, conversion from fibers into powders increases the material surface and, thus, remarkably improve their reactivity and absorbency [3,4,5]. Significantly, much more active groups are exposed on the wool powder due to its porous structure. Many applications of wool powder have been reported. For example, Wen et al. used WPs as sorbents to remove Co^2+^ from solution at appropriate pH values [4]. It can be inferred that WPs have potential applications in separation and recovery of metal ions from industrial effluents and environmental waterways [4,5]. Superfine WPs were combined with chitosan to form a membrane. The addition of WPs improved water resistance of the complex membrane and increased the dye uptake of the membrane as well [6]. Thus, it is promising to develop the applications based on powders/particles from biomass materials because of its environmentally friendly nature.

Functional nanoparticles have been combined with natural fibrous materials to fabricate functional fibers with special features, such as antibacterial, UV-blocking, and flame retarding properties. Recently, the synthesis of nanoparticles using natural fibers have attracted intensive attention of researchers and engineers, due to strong affinity, simple procedure, and environmentally friendly features. We synthesized silver nanoparticles (AgNPs) in situ on cotton fibers under basic conditions. Cotton fibers reduced silver ions to form nanoparticles due to the reducibility of cellulose fibers and acted as a support for AgNPs. The treated cotton fibers with AgNPs exhibit strong antibacterial activity and vivid colors [7]. Additionally, gold nanoparticles (AuNPs) were synthesized in situ on the surface of silk fibers. However, it is difficult to synthesize silver or gold nanoparticles on the wool fiber, which may be due to the low reducibility of bulk wool fibers. An additional reducing agent is required to prepare the metal nanoparticles on the surface of wool fibers, such as trisodium citrate [8]. WP could display high reducing activity due to its porous structure. In comparison with wool fibers, WPs possess a porous structure and a large surface area, as well as more exposed groups, which gives rise to high reactivity in addition to enhanced adsorption ability [2]. This inspired us to develop the complexes of WPs and functional nanoparticles and explore their potential applications.

Herein, in situ synthesis of AuNPs on WP was achieved by heating under the controlled pH of the reaction system. The acidic condition facilitated the reduction of Au ions in solution with WP. Factors, including the concentration of Au ions, heating temperature, and pH value, were explored. The optical properties, microstructures, and chemical composition of the AuNP-treated WP (AuNP-WP) were investigated. The obtained complexes of WP and AuNPs were applied to catalyze the reduction of 4-nitrophenol (4-NP) by sodium borohydride (NaBH_4_). Additionally, the reusability of AuNP-WP as a catalyst was estimated.

## 2. Results and Discussion

**Figure 1:**
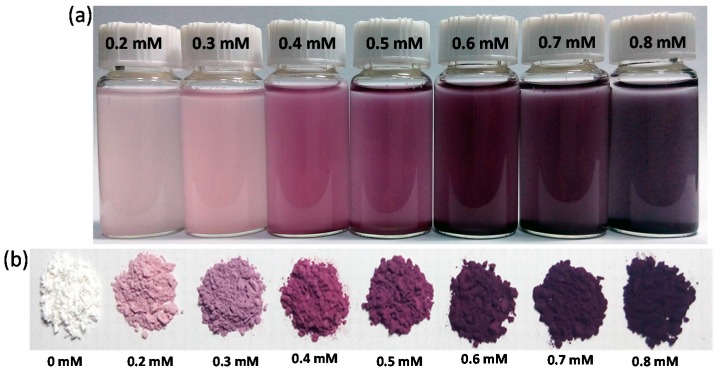
Photographs of (**a**) AuNP-WP solutions and (**b**) corresponding dry WPs for different concentrations of Au ions (0.2–0.8 mM).

Figure 1a shows the photograph of the solutions containing WP and AuNPs after heating. The color of WPs in solution changed to light red, red, purple, and dark purple from white, which reveals that AuNPs were produced from HAuCl_4_ in the presence of WP by heat treatment. The colors of the AuNP-WP solution deepened as the initial concentration of Au ions increased. It is suggested that the color of the treated WP is generated from the localized surface plasmon resonance (LSPR) optical feature of AuNPs. The WP with AuNPs was separated from solution and dried. The dry WP inherited the color of AuNPs in solution. Though the surrounding around AuNP-WP was converted to air from water, their colors did not change visibly (as shown in Figure 1b), which implies that the aggregation status of AuNPs on WP changed little during drying. These results demonstrated that WP provides a strong stabilizing effect for AuNPs synthesized in situ on WP.

**Figure 2:**
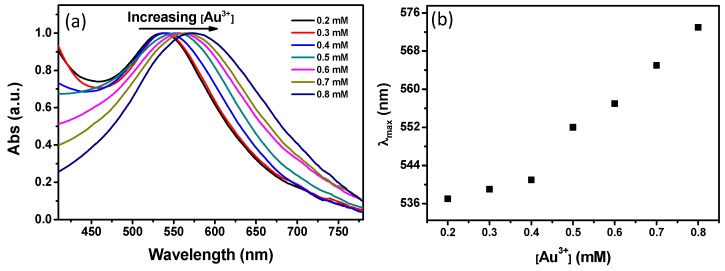
(**a**) Normalized UV-VIS diffuse reflectance absorption spectra of WP-Au corresponding to different concentrations of Au ions; and (**b**) a plot of the wavelength of the absorption peak as a function of Au ion concentration.

To further observe the optical properties of different WPs, ultraviolet-visible (UV-VIS) diffuse reflectance absorption spectra were measured. The dry WPs with AuNPs were pressed into small disks by using a pellet press die. Subsequently, UV-VIS reflectance absorption spectra were obtained from these WP disks (Figure 2a). The treated WPs displayed single absorption bands in the range of 535–575 nm. This single absorption band can be attributed to the characteristic LSPR band of AuNPs [9,10], indicating that AuNPs were synthesized in situ on WPs. The wavelength of the LSPR band of WP corresponding to 0.2 mM was located at 537 nm. The LSPR band red-shifted with an increase in the initial Au ion concentration. The WP with AuNPs from 0.8 mM of Au ions presented an UV-VIS absorption band of 573 nm. It is speculated that the red-shift of the LSPR bands may be due to aggregation of AuNPs.

**Figure 3:**
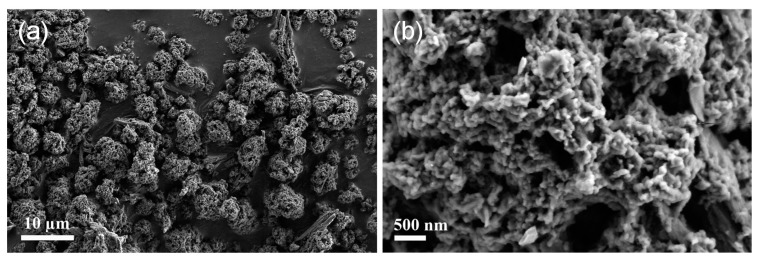
SEM images of the untreated wool powders at: (**a**) low and (**b**) high magnifications.

**Figure 4:**
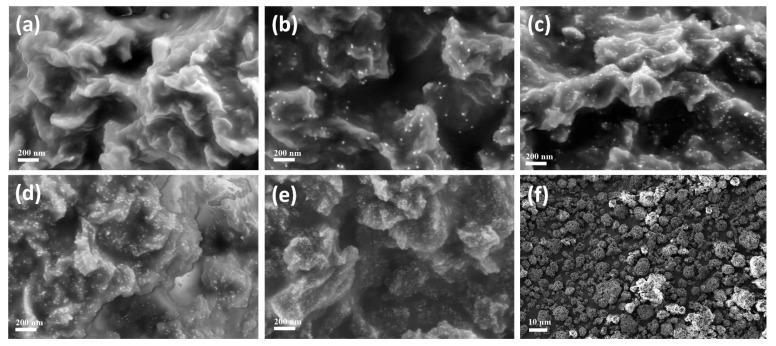
SEM images of the treated wool powders corresponding to different initial Au ion concentrations: (**a**) 0.2 mM; (**b**) 0.3 mM; (**c**) 0.4 mM; (**d**) 0.5 mM; and (**e**,**f**) 0.6 mM.

Scanning electron microscopy (SEM) was employed to observe the morphologies of WP samples. Figure 3 presents the SEM images of untreated WP. All of the wool particles appear separated in vision field and the average size of WPs was measured to be ~5 µm (Figure 3a). Porous structures can be seen from enlarged SEM image of wool particles (Figure 3b). Figure 4 shows the SEM images of WPs from different concentrations of Au ions (0.2–0.6 mM). A few AuNPs can be seen in the SEM image of AuNP-WP for 0.2 mM Au ions (Figure 4a). The number of synthesized AuNPs was limited due to low initial concentration of Au ions, which can also be inferred from the light red color of WP after heat treatment (Figure 1b). Lots of AuNPs were found in the SEM images of WPs when the Au ion concentration increased to 0.3 mM (Figure 4b). It can be seen that the amount of AuNPs was increased as the concentration of Au ions increased (Figure 4b–e). The average sizes of AuNPs on WP were measured as 22.6 ± 2.4, 13.8 ± 1.8, 12.6 ± 1.4, and 9.4 ± 0.9 nm corresponding to 0.3, 0.4, 0.5, and 0.6 mM of Au ions, respectively. The size of AuNPs decreased with an increase in Au ion concentration. According to previous reports [9,10], reducing AuNP size leads to the blue-shift of LSPR band. Therefore, red-shift of LSPR band of AuNP-WP could not result from a variation of particle size of AuNPs. Comparing the SEM images, we can find that the aggregation extent of AuNPs on the surface of WP increased when the concentration of AuNPs was increased, which is attributed to high activity of small NPs and high density of particles at high concentration of Au ions. It is possible that the mild aggregation of AuNPs led to the red-shift of LSPR band of AuNP-WP. In addition, it was noted that the heat treatment did not change morphologies of WPs in comparison with untreated WP (Figure 3a and Figure 4f), keeping porous microstructures, which is conducive to the application of complexes of WP and AuNPs.

**Figure 5:**
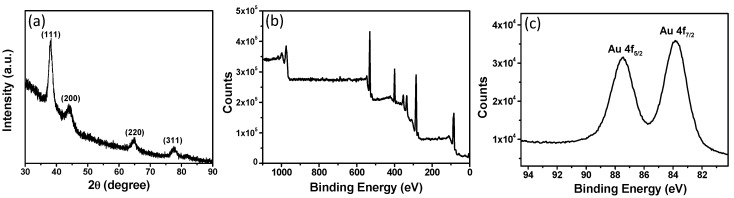
(**a**) XRD pattern of wool powder with AuNPs; (**b**,**c**) XPS spectra of wool powders after heat treatment.

Figure 5a displays the X-ray diffraction (XRD) patterns of WP after heat treatment with 0.4 mM of Au ions. Three obvious 2θ diffraction peak appeared at 38.2°, 44.3°, 64.8°, and 77.5°, which are ascribed to (111), (200), (220), and (311) crystal planes of gold [11]. The XRD result reveals that AuNPs with a face-centered cubic (fcc) crystal structure were synthesized on WP by heat. Furthermore, X-ray photoelectron spectroscopy (XPS) measurement was carried out to analyze the surface elements of WP after treatment with AuNPs. The normal components of the wool corresponding to the peaks of (O 1s), (N 1s), (C 1s), and (S 2p) are present in XPS curve (Figure 5b). The peaks of (Au 4f) appeared in the XPS data of WP after synthesis of AuNPs. In a high-resolution XPS spectrum (Figure 5c), two peaks were observed at 83.8 and 87.5 eV, which are attributed to Au 4f_7/2_ and Au 4f_5/2_ of elemental gold [12,13]. The XPS data provided a solid evidence for the presence of AuNPs on WP from the reduction of Au ions by heat treatment. Moreover, inductively-coupling plasma atomic emission spectrometry (ICP-AES) was used to determine the Au loading of different AuNP-WP samples. The Au content was measured to be 0.114%, 0.186%, 0.268%, 0.319%, 0.419%, 0.569%, and 0.608%, corresponding to the AuNP-WPs for the initial concentrations of Au ions from 0.2 to 0.8 mM, respectively. The ICP-AES data indicates the Au loading of WPs increased as an increase in the Au ion concentration of initial reaction system.

**Figure 6:**
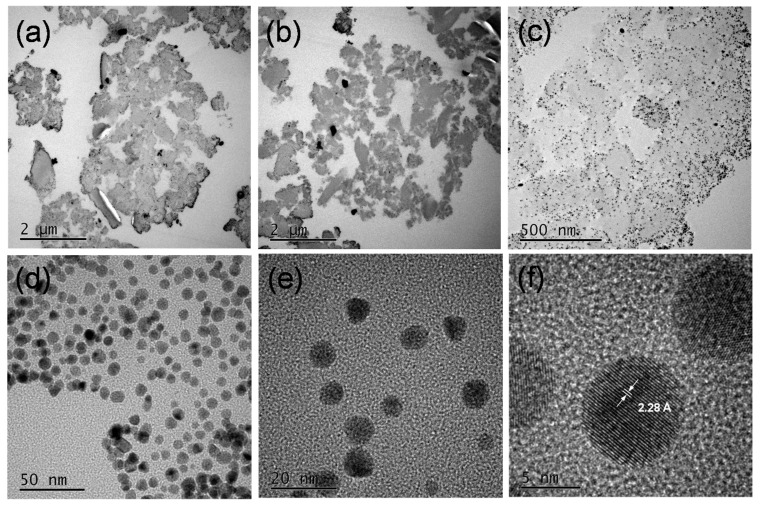
TEM images of the ultra-microtomed sections of AuNP-WP sample from 0.6 mM of Au ions at different magnifications.

**Figure 7:**
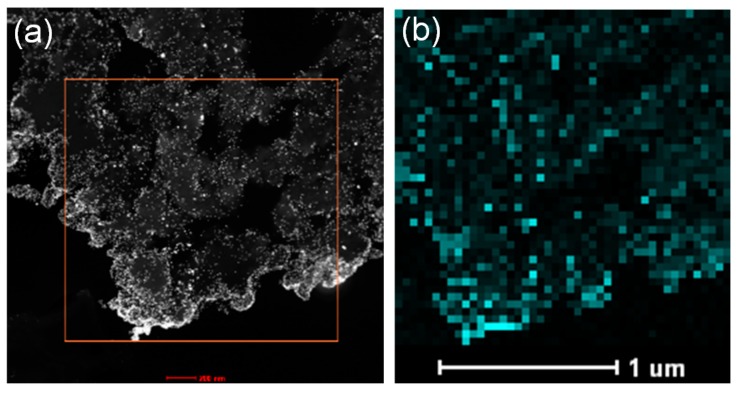
(**a**) STEM image of AuNP-WP sample from 0.6 mM of Au ions and (**b**) elemental mapping of Au from a region marked by a box in (a).

Furthermore, the ultrathin sections of wool powder with AuNPs were prepared through an ultramicrotomy technique and observed using a transmission electron microscope (TEM) to reveal the internal structures and morphologies of AuNP-WP. Figure 6 displays the TEM images of the ultra-microtomed sections of AuNP-WP sample at different magnifications. Numerous pores were seen clearly in the internal part of wool powder from the TEM image at low magnification (Figure 6a,b). The porous structures lead to large surface area, which was also demonstrated by the Brunauer–Emmett–Teller (BET) method in the previous research [2,14]. As can be found, the synthesized AuNPs distributed over the whole wool particle, which is due to the porous feature of wool powder (Figure 6c). However, the density of Au NPs on periphery of wool particles was higher than those of the internal parts. In comparison with internal parts, the external surface of wool powder interacts much readily with Au ions in solution, which results in the higher density of Au NPs on the surface wool particles. The distribution of Au NPs in internal wool powder was homogeneous, without visible aggregations (Figure 6c,d). Wool powder provides effective supporting role for as-synthesized Au NPs. High-resolution TEM images were also collected and fringes of the AuNPs can be observed (Figure 6e,f). The fringe spacing is measured to be 2.28 Å (Figure 6f), which can be ascribed to the (111) plane of fcc gold (2.35 Å) (JCPDS card No.: 04-0784). In addition, a high-angle annular dark-field scanning transmission electron microscopy (HAADF-STEM) image and the corresponding elemental mapping of Au (Figure 7) demonstrate that the Au NPs distributed over the whole wool powder after in situ synthesis of Au NPs.

**Figure 8:**
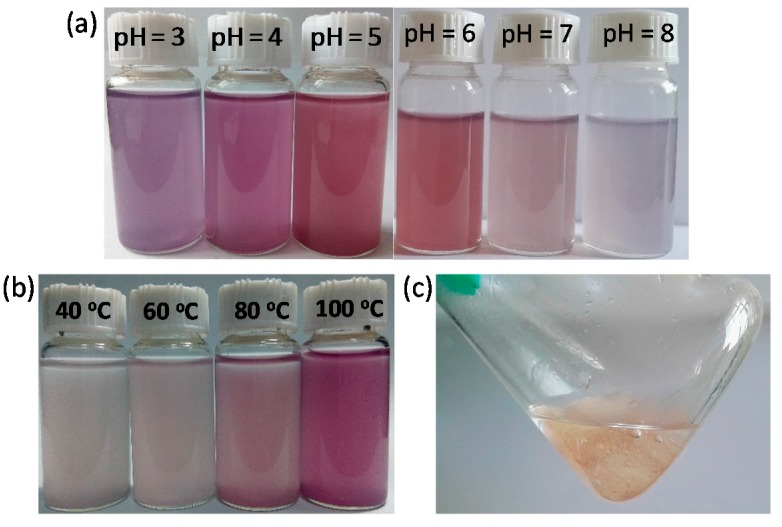
Photographs of AuNP-WP solutions obtained (**a**) at different pH values and (**b**) at different temperatures; and (**c**) photograph of solution containing wool fiber and Au ions after being heated 2 h. The concentration of Au ions was kept as 0.4 mM.

The influence of pH value on the in situ synthesis of AuNPs was investigated. The original pH value of HAuCl_4_ with 0.2~0.8 mM is around 4. Herein, the initial concentration of AuNPs was fixed as 0.4 mM, the pH values of the reaction solutions were adjusted to 3–8. After heat treatment, the solutions containing WP and Au ions at pH = 3 changed to purple (Figure 8a), suggesting the production of AuNPs. The color intensity of solutions became weak as the pH value increased to 6 from 3. It is possible that the low pH value could induce aggregation of AuNPs on WP to result in the changes of optical properties of WP with AuNPs. The solutions of WPs and Au ions changed slightly in color when the pH values of solutions were increased to 7 and 8, implying that nearly no AuNPs were synthesized at pH = 7 and 8. These results indicate that an acidic condition can facilitate the reduction of Au ions in the presence of WP by heat treatment.

**Figure 9:**
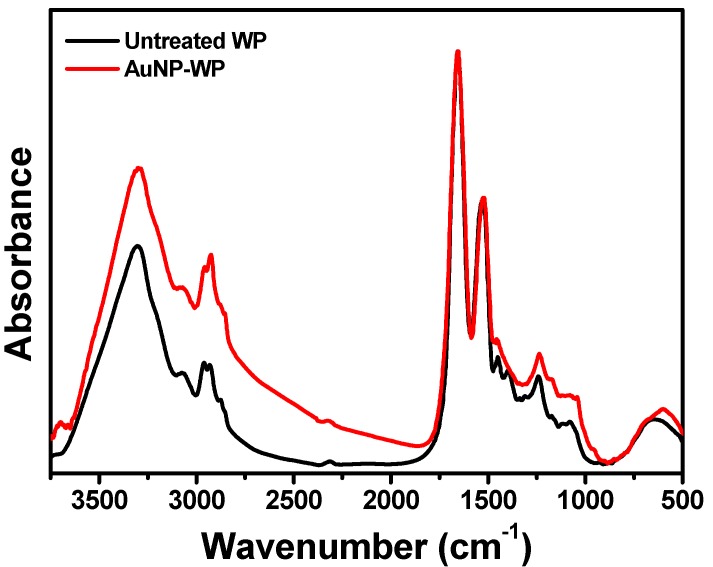
Normalized FTIR spectra of untreated wool powder and AuNP-WP from 0.06 mM of Au ions.

Heating temperature plays a vital role on the synthesis of AuNPs on WP. The reaction solution was still white after being heated for 2 h at 40 °C, implying that few or no AuNPs were produced at low temperature (Figure 8b). The colors of the solution deepened as the reaction temperature increased. A red solution was obtained when the temperature was 100 °C, revealing synthesis of AuNPs on WP (Figure 8b). It is suggested that high energy is necessary to achieve the reduction of Au ions by WP because of limited reducing ability of WP. However, significantly different from WP, bulk wool fibers could not reduce Au ions to nanoparticles in aqueous solution even after being heated for 2 h at 100 °C (Figure 8c). This testified that the transformation from fiber to powder enhanced the reactivity of wool, mainly due to the exposure of more active groups from porous microstructures. Wool as a protein material processes reaction activity, owing to containing reactive groups, including peptide bonds, side chains of amino acid residues, and disulfide crosslinks [15]. These reactive groups could act as reducing components to synthesize AuNPs under heating conditions. Fourier-transform infrared (FTIR) spectroscopy was employed to analyze the chemical structure of wool powder. In the FTIR spectrum of the untreated wool powder (Figure 9), the absorption bands 1653, 1534, and 1240 cm^−1^ are assigned to Amide I, Amide II, and Amide III, respectively [15,16]. Amide I and Amide II bands are primarily related to the C=O stretching and N–H bending, respectively. The Amide II band derives mainly from C–N stretching and N–H in-plane bending [17]. The Amide I mode of proteins at 1650–1657 cm^−1^ implies the presence of crystalline α-helix structure [15,16]. In this study, the band at 1653 cm^−1^ reveals that the wool powders contain a high proportion of amides in the α-helix conformation. The broad band around 3303 cm^−1^ could associate with the stretching vibration of N–H bond [16]. In-situ synthesis of AuNPs gave rise to the FTIR band shifting on Amide II (from 1534 to 1523 cm^−1^), Amide III (from 1240 to 1236 cm^−1^) and the broad band (from 3303 to 3297 cm^−1^) (Figure 9), which indicates that the AuNPs may interact with the amino group on wool powder.

**Figure 10:**
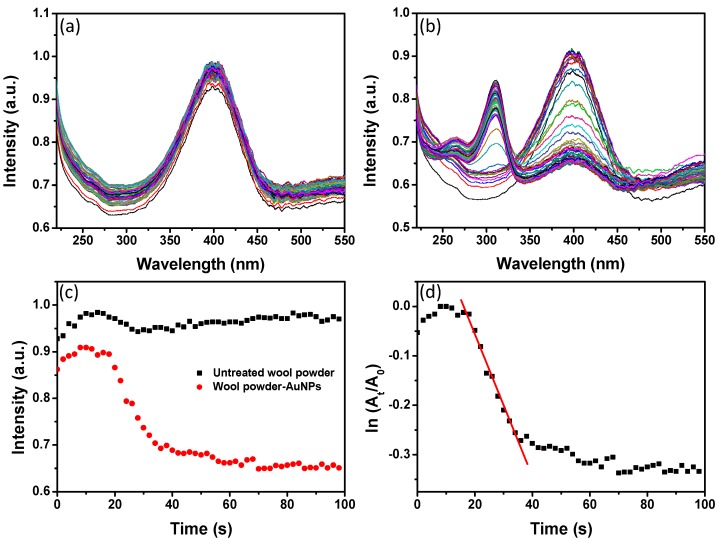
Evolution of UV-VIS absorption spectra of 4-nitrophenol solution with (**a**) untreated wool powder and (**b**) wool powder with AuNPs (from 0.06 mM of Au ions) after NaBH_4_ solution was added; (**c**) plots of absorption intensity of 4-NP (400 nm) as a function of reaction time corresponding to untreated and treated wool powders; and (**d**) a plot of ln (A_t_/A_0_) of the absorption peak at 400 nm versus time in the presence of AuNP-WP. The time interval between UV-VIS adsorption spectra was 2 s.

**Figure 11:**
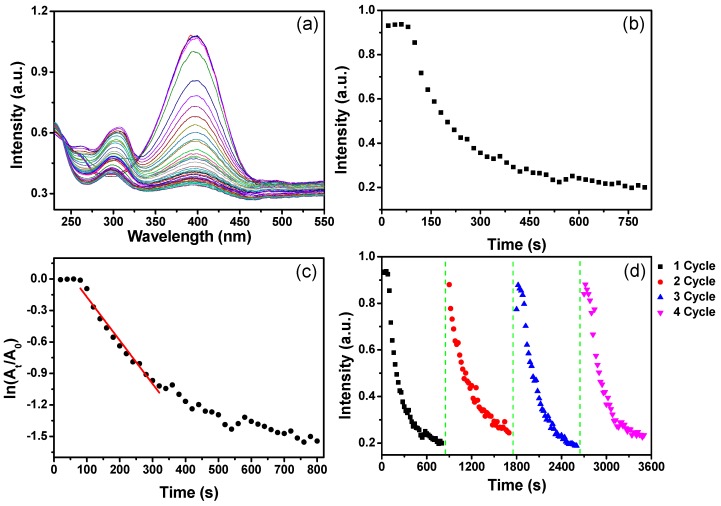
(**a**) Evolution of UV-VIS absorption spectra of 4-nitrophenol solution with wool powder with AuNPs (from 0.04 mM of Au ions) after NaBH_4_ solution was added; (**b**) corresponding plots of absorption intensity at 400 nm versus reaction time; (**c**) ln (A_t_/A_0_) of absorption peak at 400 nm as a function of reaction time; and (**d**) recycling and reuse of AuNP-WP for the reduction of 4-NP to 4-AP. The time interval between UV-VIS absorption spectra was 20 s.

AuNPs have been often used to catalyze the reduction reactions involving nitrophenols, nitroanilines, and dyes [18,19]. In the present research, the complexes of WP and AuNPs were fabricated through an in situ synthesis reaction. The porous structures of WP are beneficial to catalytic effect of AuNPs, and the microscaled particles of wool are easily separated through precipitation or centrifugation processes. WP not only provides a support for AuNPs, but also facilitates the separation of AuNPs from the catalyzed reaction system after the reaction is finished, for reuse of the catalysts. The reduction of 4-nitrophenol (4-NP) as a common catalytic model reaction was used to evaluate the catalytic activity of WP with AuNPs. The catalytic activity of the as-prepared WP with AuNPs was evaluated through monitoring UV-VIS absorption spectra of aqueous solution during the reduction of 4-NP by sodium NaBH_4_. The solution of 4-NP presented an absorption peak at 400 nm after adding NaBH_4_ solution, due to the formation of 4-nitrophenolate ions [20]. Figure 10a,b show the time-resolved UV-VIS absorption spectra of the solution containing 4-NP and NaBH_4_ in the presence of untreated WP and treated WP with AuNPs (from 0.6 mM of Au ions), respectively. For untreated WP, the UV-VIS absorption band of 4-NP changed slightly in intensity (Figure 10a), whereas the absorption intensity at 400 nm of 4-NP solution with AuNP-WP decreased dramatically with reaction time after the addition of NaBH_4_ (Figure 10b). A new absorption peak around 300 nm appeared during this process, suggesting the formation of 4-aminophenol (4-AP) [21,22]. Figure 10c displays the plots of the absorption intensity at 400 nm as a function of time corresponding to untreated and treated WPs, which can indicate the reduction rate of 4-NP by NaBH_4_. The absorption intensity at 400 nm of 4-NP solution with untreated WP did not change distinctly, suggesting the untreated WP did not show catalytic activity for reduction of 4-NP. However, the UV-VIS absorption intensity corresponding to AuNP-WP decreased dramatically, implying that the AuNP treated WP exhibited remarkable catalytic activity for reduction of 4-NP. Generally, the reduction of 4-NP is considered as pseudo-first-order kinetic reaction in the presence of excess NaBH_4_ [23,24]. Figure 10d shows a plot of ln (A_t_/A_0_) versus time for the catalytic reaction with AuNP-WP. A_t_ and A_0_ represent the absorption intensity at 400 nm at the time of *t* and the initial stage, respectively. The pseudo-first-order assumption can be testified by the linear correlation between ln (A_t_/A_0_) and time from Figure 10d. The apparent rate constant (K_app_) of the catalytic reactions can be obtained from the linear slop of ln (A_t_/A_0_) versus time and were estimated to be 1.44 × 10^−2^ s^−1^. The K_app_ values obtained from treated WPs are compared to the related results in literature for AuNPs [25,26,27]. These results manifest that WPs with in-situ synthesized AuNPs have significant catalytic activity for reduction of 4-NP. The catalytic reducing reaction rate decreased when the initial Au ion concentration of AuNP-WP sample decreased to 0.4 mM from 0.6 mM (Figure 11a,b). Whereas, the AuNP-WP from 0.4 mM of Au ions still exhibit strong catalytic activity, with an apparent rate constant of 4.14 × 10^−3^ s^−1^ (Figure 11c). In order to evaluate the reusability of the catalyst, the AuNP-WP (from 0.4 mM of Au ions) was separated from reaction system and reused in repeated reduction reactions of 4-NP. Figure 11d shows four cycles of use of AuNP-WP for reduction of 4-NP. The catalytic activity of Au NP treated wool powder did not decrease remarkably even after the fourth cycle. These results demonstrate that wool powder with AuNPs possesses high catalytic activity and good durability. Combining the porous microstructural from biomass powder and the catalytic activity from AuNPs, the complexes of AuNP-WP have potential applications in environmental pollutant treatment based on efficient adsorption and catalytic degradation.

## 3. Materials and Methods

### 3.1. Materials

Tetrachloroauric (III) acid (HAuCl_4_·3H_2_O, >99%), sodium borohydride (NaBH4, >98%), and 4-nitrophenol (4-NP) were purchased from Sinopharm Chemical Regent Co., Ltd. (Shanghai, China). All chemicals were of analytical grade and used as received.

### 3.2. Instruments

The UV-VIS diffuse reflectance absorption spectra of WPs were recorded by a Varian Cary 5000 UV-VIS-NIR spectrophotometer (Palo Alto, CA, USA) with a diffuse reflectance accessory (DRA-2500). Time-resolved UV-VIS extinction/absorption spectra were obtained with an Ocean Optics USB4000 spectrometer (Dunedin, FL, USA) and recorded using Ocean Optics SpectraSuite software. SEM measurements were performed with a Supra 55 VP field emission SEM (Zeiss, Oberkochen, Germany). A FEI Tecnai G2 F30 high-resolution transmission electron microscope (HRTEM) (Hillsboro, OR, USA) equipped with an energy-dispersive X-ray spectroscopy (EDS) detector was used to investigate the microstructure and chemical composition of AuNP-WP. The sample was embedded in resin and left to dry. The resin blocks with AuNP-WP samples were cut into ultra-thin sections (~70 nm in thickness) using a diamond knife on a Leica EM UC6 ultra-microtome machine (Buffalo Grove, IL, USA), and then collected with cooper grids for TEM measurement. XRD patterns were collected using a Bruker D8 Advance X-ray diffractometer (Madison, WI, USA) with Cu Kα radiation. XPS measurements were carried out on a Kratos XSAM800 XPS system (Manchester, UK) with Kα source and a charge neutralizer. Heating reaction was performed in a Stuart SBS40 shaking water bath (Staffordshire, UK). An IRIS Intrepid II XSP ICP-AES instrument (Thermo Fisher Scientific, Waltham, MA, USA) was employed to determine the Au loading of different AuNP-WP. A speed vacuum concentrator (Ecospin 3180C Hanil R&D, Seoul, Korea) was used to dry complexes of WP and AuNPs.

### 3.3. Preparation of Wool Powder

Wool powders were prepared through ball milling wool fibers according to the procedure in an early report [2]. Briefly, wool fibers were chopped into small snippets using a cutter mill (Pulverisette 19 from Fritsch GmbH, Markt Einersheim, Germany) equipped with a 1 mm grid. 200 g of chopped snippets in 2 L of deionized water were wet milled for 5 h in a stirred media mill (1S Attritor from Union Process, Akron, OH, USA) at a stirrer speed of 280 rpm, using milling media of yttrium-doped zirconium oxide balls (20 kg, 5 mm in diameter). Subsequently, the slurry from wet milling was spray dried (B-290 from Buchi Labortechnik AG, Flawil, Switzerland) to prepare the wool powder.

### 3.4. In Situ Synthesis of AuNPs on WPs

One hundred milliliters of HAuCl_4_ aqueous solution with different concentrations (0.2–0.8 mM) were heated to a boil in a three-necked bottle. Then, 40 mg of wool powder (WP) was added into the HAuCl_4_ solution. The color of WP changed to red, purple, or dark purple during heating. The reaction bottle was kept boiling for 30 min and cooled to room temperature. After being placed for 12 h, WP precipitated to the bottom of the bottle. Supernatant liquor was removed and sediment was centrifuged for 6 min at 6000 rpm, followed by a further centrifugation of 1000 rpm for 3 min. The resultant sediment (WP with AuNPs) was dried for 2 h in a speed vacuum concentrator.

### 3.5. Catalytic Activity

To investigate the catalytic efficiency of as-prepared WP with AuNPs, the catalytic conversion of 4-NP into 4-AP by NaBH_4_ was performed in the presence of untreated and treated WPs. In a typical experiment, 2.0 mL of 4-NP aqueous solution (5.0 × 10^−5^ M) was put into a quartz cuvette with a path length of 1 cm. Twenty microliters of WP solution (10 mg/mL) was mixed with the 4-NP solution. Subsequently, 100 µL of NaBH_4_ solution (1.0 M) was added to the mixed solution of 4-NP and WP under stirring. Meanwhile, time-resolved UV-VIS absorption spectra were recorded. The parameters of time-resolved UV-VIS absorption spectra were set as follows: integration time, 8 ms; scans to average, 10; boxcar width, 10.

## 4. Conclusions

Wool powder (WP) was used as a reducing agent to synthesize in situ gold nanoparticles (AuNPs) from Au ions. The as-synthesized AuNPs were attached to WP. The size of the AuNPs on complexes decreased as the initial concentration of Au ions increased. While the density of AuNPs on WP increased with an increase in Au ion concentration. TEM images of the ultra-microtomed sections showed that the as-synthesized Au NPs distributed over the wool particles. The porous structure led to the synthesis of AuNPs in the internal part of WP. XRD, XPS, and elemental mapping confirmed the in situ synthesis of AuNPs on WP. The acidic condition and high temperature were conducive to the synthesis of AuNPs in the presence of WP. The reactivity of WP was higher than that of wool fibers, and the fiber itself was unable to reduce Au ions to form nanoparticles. Significantly, the complex of WP and AuNPs could accelerate the reduction reaction of 4-nitrophenol, showing remarkable catalytic activity and good reusability.
